# Intercellular Adhesion Molecule 1 Promotes HIV-1 Attachment but Not Fusion to Target Cells

**DOI:** 10.1371/journal.pone.0044827

**Published:** 2012-09-06

**Authors:** Naoyuki Kondo, Gregory B. Melikyan

**Affiliations:** 1 Division of Pediatric Infectious Diseases, Emory Children's Center, Atlanta, Georgia, United States of America; 2 Children's Healthcare of Atlanta, Atlanta, Georgia, United States of America; German Primate Center, Germany

## Abstract

Incorporation of intercellular adhesion molecule 1 (ICAM-1) into HIV-1 particles is known to markedly enhance the virus binding and infection of cells expressing lymphocyte function-associated antigen-1 (LFA-1). At the same time, ICAM-1 has been reported to exert a less pronounced effect on HIV-1 fusion with lymphoid cells. Here we examined the role of ICAM-1/LFA-1 interactions in productive HIV-1 entry into lymphoid cells using a direct virus-cell fusion assay. ICAM-1 promoted HIV-1 attachment to cells in a temperature-dependent manner. It exerted a marginal effect on virus binding in the cold, but enhanced binding up to 4-fold at physiological temperature. ICAM-1-independent attachment in the cold was readily reversible upon subsequent incubation at elevated temperature, whereas ICAM-1-bearing particles were largely retained by cells. The better virus retention resulted in a proportional increase in HIV-1 internalization and fusion, suggesting that ICAM-1 did not specifically accelerate endocytosis or fusion steps. We also measured the rates of CD4 engagement, productive endocytosis and HIV-endosome fusion using specific fusion inhibitors. These rates were virtually independent of the presence of ICAM-1 in viral particles. Importantly, irrespective of the presence of ICAM-1, HIV-1 escaped from the low temperature block, which stopped virus endocytosis and fusion, much later than from a membrane-impermeant fusion inhibitor targeting surface-accessible particles. This result, along with the complete inhibition of HIV-1 fusion by a small molecule dynamin inhibitor, implies this virus enters lymphoid cells used in this study *via* endocytosis and that this pathway is not altered by the viral ICAM-1. Our data highlight the role of ICAM-1 in stabilizing the HIV-1 attachment to LFA-1 expressing cells, which leads to a proportional enhancement of the receptor-mediated uptake and fusion with endosomes.

## Introduction

HIV-1 Env glycoprotein initiates infection by fusing the viral envelope membrane with a target cell membrane. Sequential binding of Env to CD4 and coreceptors (CXCR4 or CCR5) [1]–[4] induces conformational changes in its transmembrane subunit, gp41, which promotes membrane fusion upon refolding into the six-helix bundle structure [5], [6]. Entry and fusion of cell-free HIV-1 is inefficient, whereas cell-to-cell transmission provides a much more effective platform for virus dissemination [7]–[9]. It is thought that only one out of hundreds or even thousands of cell-free virions establishes productive infection [10]–[15]. However, accumulating evidence suggests that the apparent low efficiency of HIV-1 infection is primarily due to poor binding to target cells and not to an inherently low specific infectivity [16]–[18]. More importantly, the majority of viruses detach from the plasma membrane before undergoing endocytosis and/or fusion [18], [19]. Thus, stable adhesion to cells is emerging as an essential factor in HIV-1 entry.

HIV-1 particles are known to incorporate a number of host proteins that play a role in virus entry and replication [20]–[28]. The intercellular adhesion molecule 1, ICAM-1 (also known as CD54) is expressed by endothelial and immune cells and is involved in several important immunological events, such as activation of CD8^+^ T cells [29], signaling between lymphoid cells [30], [31], and trans-endothelial migration of leukocytes [32]–[34]. ICAM-1 is a specific ligand for LFA-1 (lymphocyte function-associated antigen-1), which is an integrin-like protein expressed by immune cells [35]–[37]. Importantly, ICAM-1 is selectively recruited into HIV-1 particles [22], [24], [28], [38], apparently through interactions between the cytoplasmic domain of ICAM-1 and immature HIV-1 Gag [39]. The virus-incorporated ICAM-1 markedly enhances HIV-1 infection of cells expressing LFA-1 by promoting the virus binding and internalization [40]–[45]. Moreover, antibodies known to increase the affinity of LFA-1 to ICAM-1, such as MEM83 and NKI-L16, further enhance the infectivity of ICAM-1-bearing viruses in lymphoid cell lines and in peripheral blood mononuclear cells (PBMCs) [43]. Additional evidence supporting the role of virus-incorporated ICAM-1 in HIV-1 infection include the diminished neutralizing activity of antibodies against Env [41], [46], [47] and the ability of ICAM-1 antibodies to block virus entry [40]–[42]. Together, these findings imply that ICAM-1/LFA-1 interactions facilitate HIV-1 infection by enhancing the virus-cell binding.

In spite of the marked enhancing effect of ICAM-1 on HIV-1 binding and infection of T cell lines and primary CD4^+^ T cells [40], [41], [43], [44], the virus fusion with primary CD4^+^ T cells appears to be only slightly (1.2-fold) enhanced by this adhesion molecule [44]. To dissect the role of ICAM-1 in HIV-1 entry, we examined its binding to and fusion with lymphoid cells using a direct virus-cell fusion assay. ICAM-1 increased the virus binding to target cells in LFA-1- and temperature-dependent manner. We found that ICAM-1-independent binding was reversible, as the majority of virions were lost from cells, whereas ICAM-1/LFA-1 interactions prevented virus dissociation from cells without significantly altering the probability of virus internalization or fusion. Measurements of the rate of HIV-1 binding to CD4, productive endocytosis and fusion with endosomes did not reveal significant differences between viruses bearing and lacking ICAM-1. Together, these data suggest that ICAM-1 promotes HIV-1 infection by stabilizing the virus adhesion to LFA-1-positive cells without specifically enhancing the virus uptake or subsequent fusion with endosomes.

## Results

### ICAM-1 promotes HIV-1 binding and fusion with LFA-1 expressing cells

**Figure 1:**
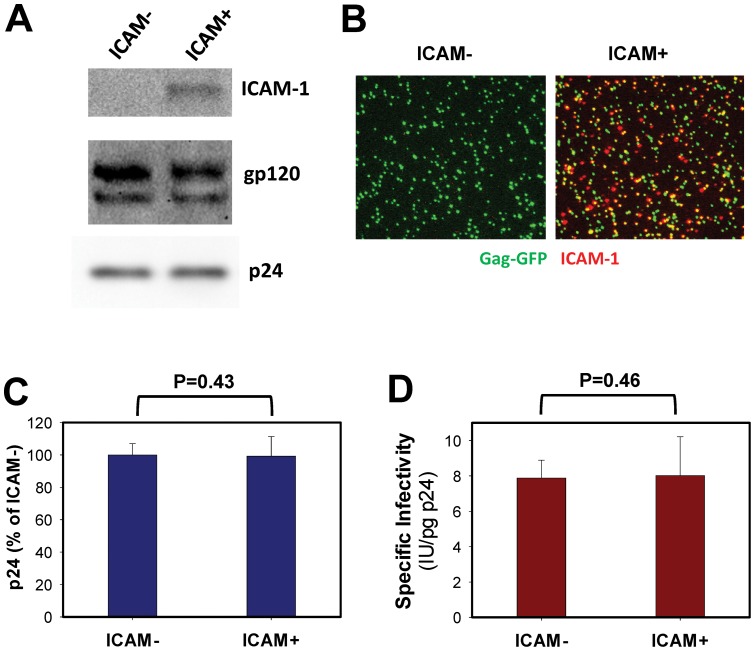
ICAM-1 incorporation into HIV-1 pseudoviruses does not diminish specific infectivity. (A) Western blotting analysis of purified HXB2 pseudoviruses lacking and containing ICAM-1 (ICAM^−^ and ICAM^+^, respectively). Equal amounts of p24 were analyzed for ICAM-1 and Env incorporation and proteolytic cleavage of Env. (B) Immunostaining of ICAM^−^ or ICAM^+^ HXB2 pseudotypes labeled with Gag-GFP (green). Viruses were immobilized onto a coverslip and incubated with anti-ICAM-1 antibody followed by anti-mouse Cy5-conjugated antibody (red). Colocalization of GFP and ICAM-1 staining (yellow) shows incorporation of ICAM-1 into virions. (C) Production of HXB2 pseudoviruses lacking or containing ICAM-1 by transfected 293T cells. The concentration of p24 in extracellular medium was determined by ELISA. (D) Viral titer for ICAM^−^ and ICAM^+^ viruses was determined on TZM-bl cells (negative for LFA-1) using β-Gal assay. Specific infectivity was calculated by dividing the viral titer (infectious units/ml) to the p24 content (pg/ml). Data points are means and SEM for three independent experiments performed in triplicate.

Pseudoviruses expressing HIV-1 HXB2 Env and lacking or bearing ICAM-1 in their membrane (hereafter referred to as ICAM^−^ and ICAM^+^, respectively) were produced by transient transfection of 293T cells. The incorporation of ICAM-1 and HIV-1 Env glycoprotein into virions was verified by immunoblotting (Fig. 1A). The ICAM-1 incorporation into virions did not appear to compete with incorporation, proteolytic processing or stability of Env, as evidenced by similar intensities of gp160 and gp120 bands for control and ICAM^+^ viruses. Specific incorporation of ICAM-1 into the viral membrane was further verified by immunostaining viruses containing Gag-GFP with antibodies against ICAM-1 (Fig. 1B). Whereas ICAM^−^ particles were not recognized by these antibodies, ∼50% of GFP-positive (green) viruses contained detectable amount of ICAM-1 (red). ICAM-1 expression in producer cells did not affect the virus production, as measured by the amount of p24 in extracellular medium (Fig. 1C), or their ability to infect LFA-1-negative TZM-bl indicator cells (Fig. 1D).

**Figure 2:**
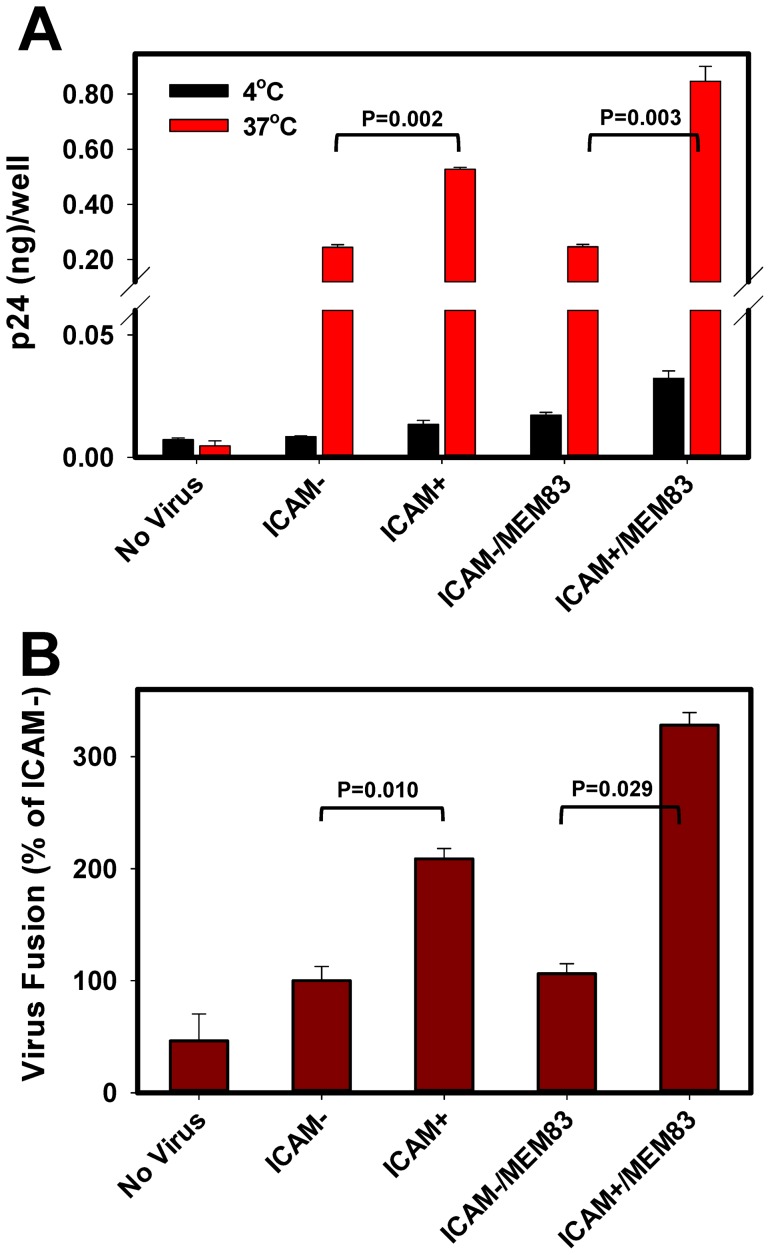
ICAM-1- and temperature-dependence of HIV-1 pseudovirus binding and fusion with PM-1 cells. (A) Cells were either pretreated with anti-LFA-1 antibody MEM83 for 30 min at 37°C or left untreated. Five ng of ICAM^+^ or ICAM^−^ viruses were incubated with 0.5·10^5^ cells in a V-bottom 96-well plate for 1.5 h, either at 4°C or at 37°C. Free viruses were removed by washing, and the amount of cell-associated particles in each well was determined by p24 ELISA. (B) Equal amounts of ICAM^−^ and ICAM^+^ viruses were incubated with cells at 37°C for 1.5 h, and the extent of fusion was measured by the BlaM assay. Data are means and SEM from three independent experiments.

We next assessed the effect of ICAM-1 on the virus binding to PM-1 cells expressing LFA-1 [43]. Virus binding to these cells at 4°C was extremely inefficient, although ICAM^+^ viruses bound somewhat better than particles lacking ICAM-1 (Fig. 2A, black bars, P = 0.039). Only ∼0.2% of viral p24 attached to cells within 90 min in the cold. However, the HIV-1 binding (and likely the virus uptake) increased dramatically at 37°C (Fig. 2A, red bars). Under these conditions, ∼4% of ICAM^−^ and ∼10% of ICAM^+^ viruses from inoculum bound to cells. LFA-1 is known to exist in closed “inactive” and open “active” conformations, which bind the ICAM-1 ligand with low and high affinity, respectively [48], [49]. To increase the affinity of ICAM-1/LFA-1 interactions, we stabilized the high affinity conformation of LFA-1 by pretreating the cells with the anti-LFA-1 antibody MEM83 [43], [50]. Whereas the ICAM^+^ virus binding in the cold was modestly enhanced upon LFA-1 activation (P = 0.013), this effect was much more significant (P = 0.002) at 37°C. Thus, in agreement with previous studies [40], [41], [43], [44], [51], ICAM-1 promoted HIV-1 binding to lymphoid cells in LFA-1- and temperature-dependent manner. While ICAM-1 similarly increased HIV-1 adhesion and fusion with other T cell lines (Fig. S1), this effect was more pronounced for PM-1 cells. The latter cells were therefore utilized throughout this study.

The ICAM-1 effect on HIV-1 fusion was next evaluated, using the β-lactamase (BlaM) assay which measures transfer of the viral core-associated enzyme into the cytoplasm [52]. Equal amounts of ICAM^+^ and ICAM^−^ viral inocula (based on the p24 content) were added to PM-1 cells and incubated for 90 min at 37°C. The resulting BlaM signal was ∼2-fold greater for ICAM^+^ viruses compared to control viruses for untreated cells and up to 4-fold greater for cells pretreated with MEM83 (Fig. 2B). Since the effect of ICAM-1 on HIV-1 fusion paralleled its effect on the virus binding (Fig. 2A and B), ICAM-1 appears to facilitate HIV-1 fusion with PM-1 through promoting the virus attachment and/or uptake.

### HIV-1 retention on the cell surface but not endocytosis is increased by ICAM-1

**Figure 3:**
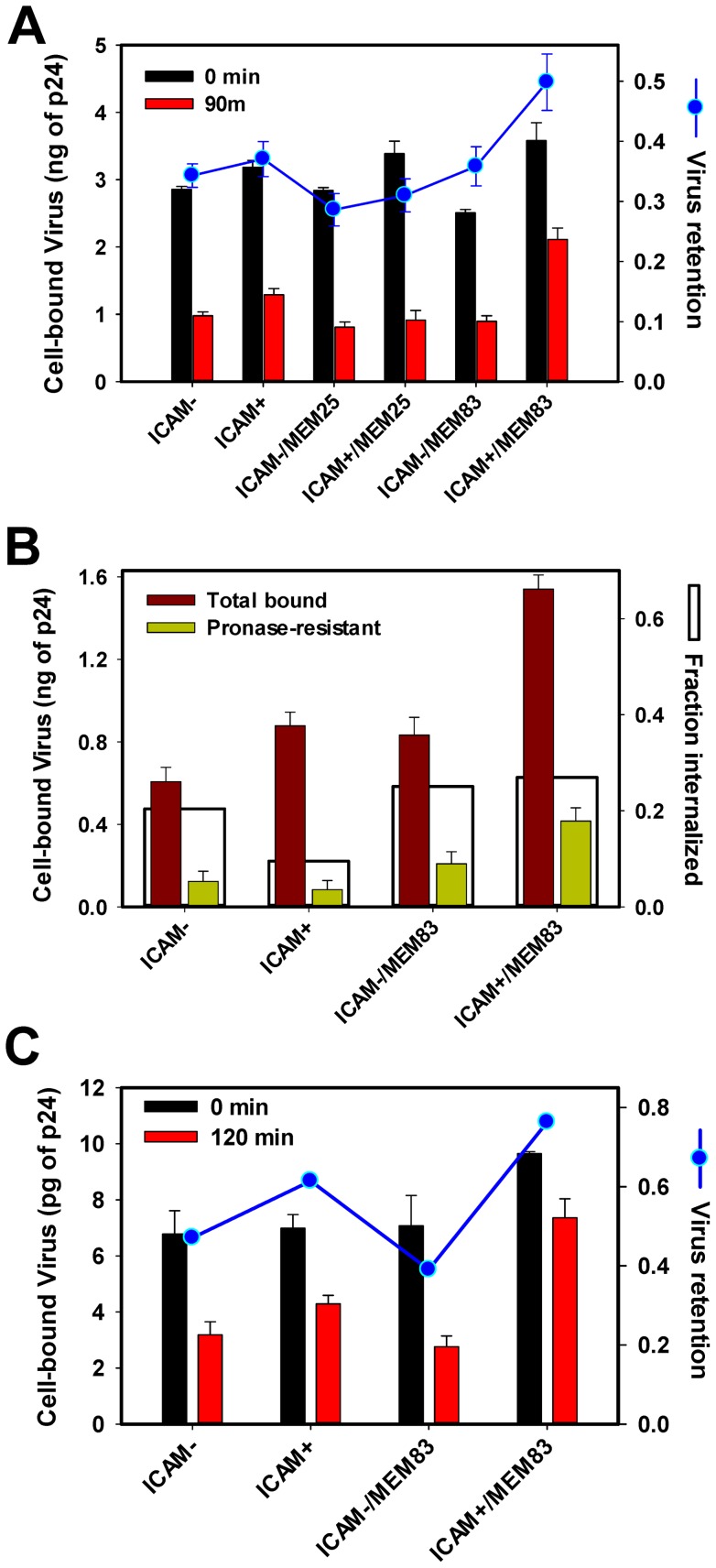
Effect of ICAM-1 on virus attachment to cells aided by centrifugation in the cold. PM-1 cells (1·10^5^) were adhered to poly-lysine-coated 96-well plates. Immobilized cells were pre-treated with MEM25 or MEM83 antibodies or left untreated. Eight ng of either ICAM^+^ or ICAM^-^ viruses were added to cells and centrifuged at 4°C for 30 min. (A) After virus binding by spinoculation, cells were washed, and the amount of p24 was measured either immediately (0 min) or after incubation at 37°C for 1.5 h (90 min). Blue symbols show the ratio between the cell-associated virus after incubation at 37°C and immediately after spinoculation. (B) To determine the fraction of internalized viruses following the pre-binding step in the cold and incubation for 1.5 h at 37°C, cells were treated with 2 mg/ml pronase for 30 min on ice or left untreated. Cells were washed, and the remaining p24 was measured by ELISA. White bars are obtained by normalizing the pronase-resistant p24 signal to the amount of virus associated with untreated cells. (C) Same as in panel A, but using HIV-1 TYBE pseudoviruses. After ICAM^−^ or ICAM^+^ viruses (1 ng of p24) were spinoculated onto cells in the cold, cells were washed and the amount of p24 was measured either immediately (0 min) or after incubation at 37°C for 2 h (120 min). Blue circles show the ratio between the cell-associated virus after incubation at 37°C and immediately after spinoculation. Data are means and SEM from a representative experiment performed in triplicate.

To discern the ICAM-1 effect on virus attachment and uptake, the entry process was synchronized by pre-binding HXB2 pseudoviruses to cells in the cold, washing off free viruses, and raising the temperature. Since binding to PM-1 cells in the cold was very inefficient (Fig. 2A), cells were first adhered to poly-lysine-coated plates and centrifuged with the virus. This protocol referred to as spinoculation [17] is known to facilitate virus binding. Centrifugation at 4°C dramatically increased the amount of cell-associated (and perhaps plastic-adhered) p24 in all samples compared to incubation at 4°C in the absence of centrifugal force (Figs. 2A and 3A). The enhancing effect of spinoculation was virtually independent of the viral ICAM-1(Fig. 3A, P = 0.206), but the LFA-1-activating antibody MEM83 selectively enhanced the binding of ICAM^+^ viruses under these conditions (P = 0.011). Interestingly, most cell-bound viruses were lost after incubation at 37°C for 90 min (Fig. 3A, red bars), whereas MEM83-pretreated cells retained 50% of initially attached virions. Pre-treatment of cells with MEM25, an antibody that blocks ICAM-1/LFA-1 interactions [50], [53], eliminated the enhancing effect of ICAM-1 on the virus retention by cells, thus demonstrating the specificity of ICAM-1-mediated virus attachment. In summary, virions bound to cells *via* spinoculation in the cold were readily lost upon further incubation at 37°C. The ICAM-1/LFA-1 interactions effectively counteracted the virus loss.

To determine whether the enhanced retention of ICAM^+^ viruses at elevated temperature was due to a more efficient virus uptake, particles remaining on the cell surface after incubation at 37°C for 90 min were stripped with pronase, and the amount of remaining p24 was determined by ELISA. We found that only a small amount of viruses was internalized by PM-1 cells under these conditions and that the HIV-1 uptake was virtually independent of the presence of ICAM-1 (P = 0.06, Fig. 3B). In parallel experiments, the total amount of cellular proteins was measured to rule out cell loses as a result of protease treatment (Fig. S2). Only after pretreatment of cells with MEM83 the amount of internalized ICAM^+^, but not ICAM^−^ viruses increased significantly (P = 0.002). However, this increment in the virus uptake was proportional to the total amount of cell-associated p24 after incubation at 37°C (Fig. 3B), implying that the fraction of internalized viruses was not modulated by ICAM-1/LFA-1 interactions. This point is illustrated by normalizing the amount of pronase-resistant virus to the total amount of cell-associated p24 (Fig. 3B, open bars). In other words, ICAM-1/LFA-1 interactions appear to prevent HIV-1 detachment from the surface of PM-1 cells without significantly accelerating virus endocytosis.

Next, we asked whether the ICAM-1 effect on HXB2 pseudovirus binding was modulated by the Env glycoprotein. Pseudoviruses bearing X4-tropic Env derived from a clinical isolate, TYBE [54], either containing or lacking ICAM-1, were prepared and tested. The nearly equal specific infectivity of ICAM^+^ and ICAM^−^ viruses in TZM-bl cells (Fig. S2) shows that incorporation of ICAM-1 does not compromise the virus' ability to establish productive infection. Similar to HXB2 pseudotypes, TYBE pseudoviruses tended to detach from cells following spinoculation in the cold and incubation at 37°C. However, ICAM^+^ particles were better retained by cells than control particles (Fig. 3C, P = 0.014). Virus binding in the cold and retention at elevated temperature were further enhanced after pretreatment of PM-1 cells with MEM83 (Fig. 3C, P = 0.015). In fact, as much as 75% of ICAM^+^ TYBE virions bound to pretreated cells in the cold remained attached after 2 h at 37°C.

### Stabilization of HIV-1 binding to cells results in proportional enhancement of fusion

**Figure 4:**
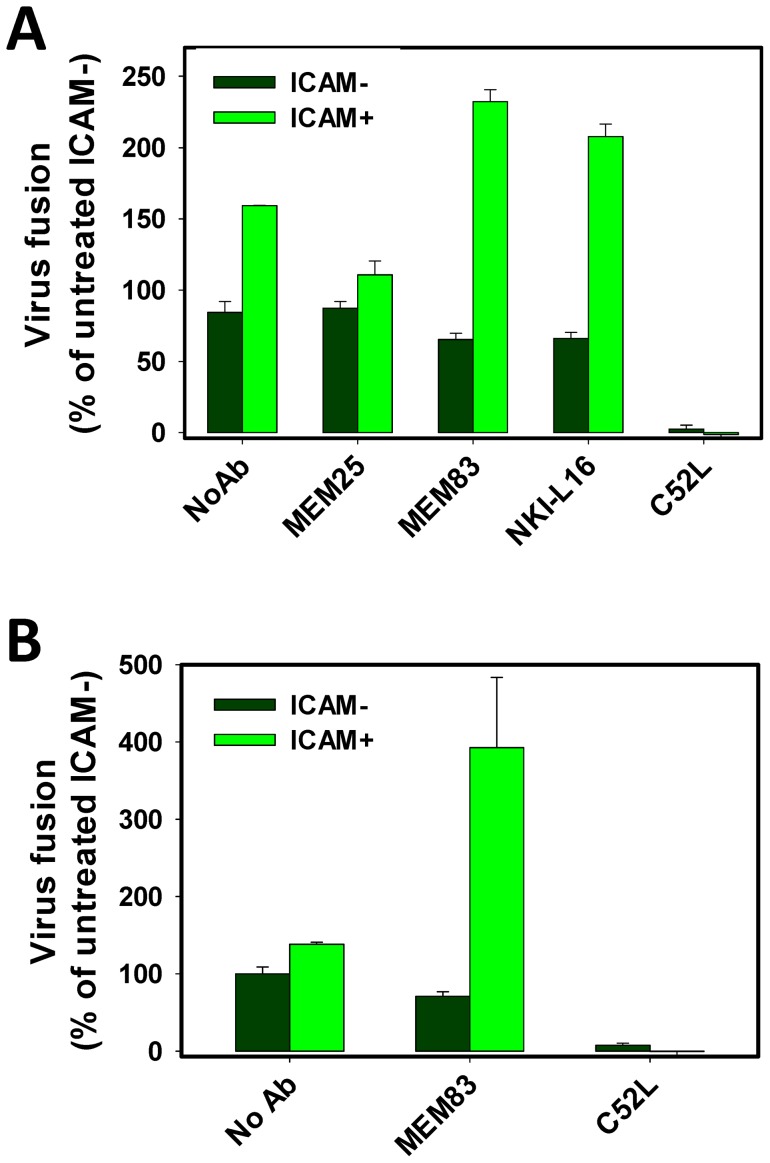
ICAM-1 promotes HIV-1 fusion. (A) Fusion of ICAM^+^ and ICAM^−^ HXB2 pseudoviruses following their pre-binding to PM-1 cells by spinoculation in the cold and incubation at 37°C for 1.5 h. Cells were either untreated or pretreated with anti-LFA-1 antibodies MEM25, MEM83 or NKI-L16 prior to inoculation. (B) PM-1 cells were pretreated with 3 µg/ml of MEM83 antibody. TYBE pseudoviruses (0.5 ng of p24) were bound to PM-1 cells by spinoculation. Cells were washed and incubated at 37°C for 2 h in the presence of MEM83. MEM83 promoted significant increase in the fusion activity compared to untreated control (P = 0.013). Data in panels A and B are means and SEM from representative experiments done in triplicate. Control wells in A and B were incubated in the presence of 1 μM C52L to block virus fusion.

We next examined whether better retention of ICAM^+^ viruses by cells could promote HIV-1 fusion. Cells were spinoculated with viruses in the cold, washed and incubated at 37°C. As shown in Fig. 4A, the same amount of ICAM^+^ inoculum produced a greater BlaM signal compared to ICAM^−^ viruses (P = 0.005). This effect was similar to augmentation of fusion by viral ICAM-1 observed upon direct incubation with cells at 37°C without spinoculation (Fig. 2B). The fusion-enhancing effect of ICAM-1 was virtually eliminated after pretreatment of PM-1 cells with the inhibitory MEM25 antibody (P>0.10) [50], [53]. By contrast, the LFA-1-activating antibodies, MEM83 and NKI-L16, further promoted fusion of ICAM^+^, but not of ICAM^−^ viruses (P = 0.002 and 0.001, respectively, Fig. 4A). Since the increment in ICAM^+^ fusion with MEM83-pretreated cells (∼4-fold) was proportional to the amount of cell-associated virus at the end of incubation at 37°C (Fig. 3A, red bars and Fig. 4A), it appears that the better retention of these viruses by cells could be the major reason for their increased fusion activity (Fig. 3B).

Fusion of TYBE pseudovirus with PM-1 cells was also significantly augmented by ICAM-1 (P = 0.020), but this effect was markedly (5-fold, P = 0.019) enhanced upon pretreating the cells with MEM83 (Fig. 4B). Together, our data support the notion that ICAM-1 stabilizes HIV-1 attachment to LFA-1 expressing cells and that better virus retention proportionally facilities downstream endocytosis and fusion events. Since the enhancing effect of ICAM-1 on TYBE pseudovirus retention (Fig. 3) was not as strong as its effect on fusion (Fig. 4), it is possible that ICAM-1/LFA-1 interactions could also facilitate late steps of fusion.

### ICAM-1 does not alter the kinetics or sites of HIV-1 fusion

**Figure 5:**
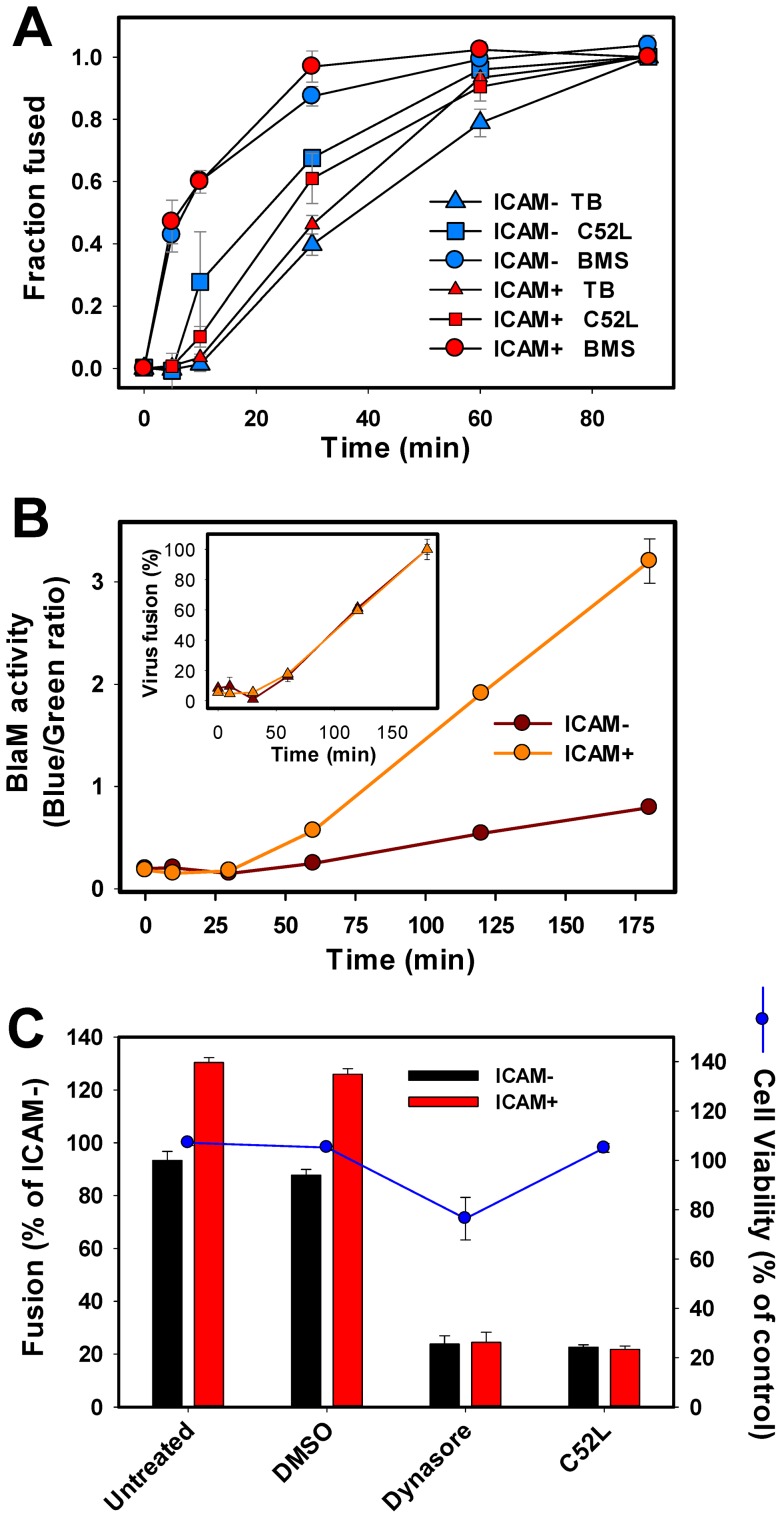
ICAM-1 does not significantly alter the kinetics of key steps of HIV-1 fusion with PM-1 cells. (A) Cells were either pretreated with the MEM83 antibody or mock-treated prior to centrifuging with equal amounts of ICAM^−^ and ICAM^+^ HXB2 pseudoviruses for 30 min at 4°C. Kinetics of entry and fusion of ICAM^−^ (blue symbols) and ICAM^+^ (red symbols) viruses upon incubation with cells at 37°C was measured by the BlaM assay. Fusion was stopped at indicated times by adding BMS-806 (10 μM), C52L (1 μM) or by lowering the temperature (temperature block, TB). The average values and SEM from three independent experiments performed in triplicate are shown. (B) Kinetics of ICAM^+^ and ICAM^−^ HXB2 pseudovirus fusion upon incubating the viruses (0.5 ng p24) and 1·10^5^ PM-1 cells directly at 37°C. Fusion was stopped at indicated times by adding 1 µM C52L, and the resulting BlaM signal was measured as the ratio of blue and green fluorescence (see Materials and Methods). *Inset*: Normalized kinetics of ICAM^+^ and ICAM^−^ virus fusion with PM-1 cells. (C) Inhibition of HXB2 pseudovirus fusion by a dynamin inhibitor, dynasore. Immobilized cells were spinoculated with viruses, washed and incubated for 1.5 h at 37°C in HBSS alone or in buffers containing either 0.1% DMSO (vehicle) or 80 μM dynasore. As a negative control, fusion was carried out in the presence of 1 µM C52L. Cell viability (blue circles) was measured by the MTS assay, as described in Materials and Methods. Dynasore significantly (P = 0.01) inhibited ICAM^+^ virus fusion with PM-1 cells.

We next sought to examine the effect of ICAM-1 on the HIV-1 progression through key steps of fusion and to assess the sites of the virus entry into PM-1 cells. Virus binding to cells was aided by centrifugation in the cold, and fusion was synchronously initiated by shifting to 37°C. Fusion was stopped at varied time points by adding high concentrations of fusion inhibitors targeting distinct steps of the fusion reaction, as described in [16], [55], [56]. HIV-1 binding to CD4 was evaluated using a small molecule inhibitor BMS-806 [57], [58]. The virus escape from inhibition by the C52L peptide, which blocks the gp41 six-helix bundle formation [59], may reflect the rate of virus fusion at the cell surface (if this pathway is operational in PM-1 cells). Our data imply, however, that HIV-1 enters these cells from endosomes and that, therefore, acquisition of resistance to C52L occurs through productive endocytosis [55], [56], [60], [61]. Finally, the rate of HIV-endosome fusion was measured by lowering the temperature (referred to as the temperature block, TB) at indicated time points and thereby stopping the virus uptake and fusion [16], [55]. Incubation of viruses and cells at 37°C resulted in sequential acquisition of resistance to BMS-806, C52L, and TB (Fig. 5A), in full agreement with our previous results [16], [55], [56]. The presence of ICAM-1 in viral particles had no significant effect on the virus progression through intermediate steps of fusion. The fact that the kinetics of virus escape from BMS-806 was not affected by ICAM-1 suggests that this molecule neither promoted nor interfered with Env-CD4 binding.

It has been previously reported that ICAM-1/LFA-1 interactions increase the kinetics of infection of the Jurkat-derived cells [40]. We therefore assessed the kinetics of HIV-cell fusion using a conventional protocol whereby viruses are mixed with cells and incubated at 37°C. Virus entry/fusion was stopped by adding a high concentration of C52L at indicated times. After prolonged incubation, ICAM^+^ viruses produced a considerably greater BlaM signal compared to ICAM^−^ viruses (Fig. 5B). However, for both viruses, fusion was detected after a pronounced lag of about 30 min and the kinetics of fusion was virtually identical (Fig. 5B, inset). Thus, ICAM-1 did not accelerate the virus binding and/or fusion with PM-1 cells either after HIV-1 pre-binding to cells or upon direct incubation at 37°C. The much slower kinetics of fusion in the latter format implies that HIV-1 binding to PM-1 cells is the rate-limiting step for entry and fusion and that ICAM-1 does not considerably accelerate this step.

We have previously shown that the delayed kinetics of HIV-1 escape from TB relative to resistance to C52L likely reflects the time interval between virus uptake and fusion with endosomes [55]. The delay between the C52L and TB kinetics of ICAM^+^ virus fusion (Fig. 5A) supports the notion that these viruses enter PM-1 cells through an endocytic route. Further evidence supporting entry of ICAM^+^ viruses through endocytosis was obtained using a small molecule dynamin inhibitor, dynasore [62]. Dynasore abrogated fusion of ICAM^−^ and ICAM^+^ viruses (Fig. 5C) without severely reducing the cell viability (blue circles). These findings support the notion that viruses bearing or lacking ICAM+ enter lymphoid PM-1 cells *via* endocytosis followed by fusion with endosomes [55].

## Discussion

ICAM-1/LFA-1 interactions play an essential role in immune activation through stabilization of immunological synapse [63], as well as in cell-cell transmission of HIV-1 through their involvement in the formation of virological synapses [64]. ICAM-1/LFA-1 interactions also appear to drive HIV-1 transmission from infected CD4+ T cells to trophoblasts [65] and from human hepatoma cells to CD4+ T cells [66]. In addition, promotion of HIV-1 Env-mediated syncytia formation by LFA-1 [67] suggests that this adhesion molecule could play a role in virus fusion. A series of publications by the Tremblay laboratory has nicely delineated the effect of ICAM-1 on HIV-1 binding, uptake and infection in lymphoid cells expressing LFA-1 [39], [40], [42]–[46], [53], [68], [69]. These studies provided compelling evidence for the enhancement of both binding and productive entry of HIV-1 into T cells by the host cell-derived ICAM-1 incorporated into virions. These experiments largely relied on the amount of p24 bound to or internalized by target cells under physiological conditions allowing for both the virus uptake and fusion.

In this study, we sought to dissect the effects of the viral ICAM-1 on HIV-1 binding, uptake and fusion, using a viral content transfer assay. Our data confirmed the enhancing effect of ICAM-1/LFA-1 interactions on HIV-1 binding and downstream endocytosis and fusion events. However, the virus uptake and fusion increased proportionally to the enhanced HIV-1 binding to target cells, suggesting that this ligand may not facilitate post-binding steps of entry beyond promoting the virus absorption. Our results thus highlight the role of ICAM-1 in reducing HIV-1 dissociation from lymphoid cells (Fig. 3), a process that appears to limits productive infection of these cells and engineered cell lines [18], [19].

Additional novel findings of this study include the demonstration that ICAM-1-bearing virions progress through intermediate steps of fusion at the same rate as control viruses. The invariant rate of CD4 engagement by ICAM^−^ and ICAM^+^ viruses (Fig. 5A) argues against the beneficial effect of LFA-1 signaling and the formation of LFA-1 clusters enriched in CD4 and possibly of CXCR4 [53] on HIV-1 fusion. Note, however, that, in order to separate the virus binding step from downstream endocytosis and fusion, we attached pseudoviruses to cells by spinoculation in the cold. This protocol may not permit high-affinity virus-LFA-1 interactions or LFA-1 clustering prior to initiating fusion by raising the temperature. Under these conditions, ICAM-1/LFA-1 interactions did not alter the preferred HIV-1 entry pathway into lymphoid cells which likely occurs through endocytosis [55], [56]. Two lines of evidence support this conclusion. First, fusion of ICAM^+^ viruses became resistant to low temperature after a considerable delay following the virus escape from a membrane-impermeant peptide targeting surface-accessible viruses (Fig. 5A). Second, ICAM^+^ viruses were as sensitive to a small molecule dynamin inhibitor, dynasore, as control viruses (Fig. 5C).

While our data are in agreement with the reported role of ICAM-1 in HIV-1 binding/infection [40], [41], [44], the differences between ICAM^+^ and ICAM^−^ viruses were more modest that those observed previously. Of note, experiments using pseudoviruses bearing a primary TYBE Env showed a somewhat more potent enhancement of the virus binding and fusion by ICAM-1 compared to HXB2 pseudotypes. Overall, however, the increment in the ICAM^+^ virus fusion appears to be primarily due to the enhanced binding and retention of these viruses by target cells. A relatively modest enhancement of pseudovirus binding, uptake and fusion by ICAM-1/LFA-1 interactions in our study compared to the earlier report [44] could be due to differences in viral preparations, including the amount of incorporated ICAM-1. In addition, our experiments employed a considerably smaller amount of viral inocula compared to the study by Tardif and Tremblay [44]. In light of recent finding that HIV-1 Env can directly activate LFA-1 [70], it is possible that a large number of virions bound per cell could promote stronger LFA-1 signaling and formation of high-affinity LFA-1 clusters, which have been proposed to facilitate HIV-1 infection [53]. Considering the highly inefficient binding and infection of target cells by cell-free HIV-1, even a modest improvement in the virus attachment could strongly promote virus replication *in vivo*.

It remains to be determined whether or not ICAM-1/LFA-1 interactions play a role in facilitating HIV-1 transmission through the virological synapse beyond stabilizing the cell-cell junction and concentrating CD4 and coreceptors [65], [71]–[73]. It is also not known whether viral ICAM-1 is involved in the proposed endocytic entry route of HIV-1 following its transmission across the virological synapse [74], [75]. Studies of ICAM^+^ virus binding and entry into primary CD4^+^ T cells could provide a simple model for complex interactions within the virological synapse.

## Materials and Methods

### Cells, plasmids and reagents

TZM-bl cells expressing CD4 and both or coreceptors, CXCR4 and CCR5, as well as the LTR-driven β-galactosidase cassette [76] and CD4^+^ T cell line, PM1 (donated from Dr. Marvin Reitz, [77]) were obtained from the NIH AIDS Research and Reference Reagent Program (ARRRP). Dulbecco's Modified Eagle Medium (DMEM), RPMI-1640, Hanks' balanced salt solution with calcium and magnesium (HBSS), fetal bovine serum (FBS), phosphate-buffered saline (PBS), Cell Stripper and G418 sulfate were purchased from Corning-Cellgro (Richmond, VA). Anti-LFA-1 antibodies MEM25 and MEM83 were from Thermo Scientific (Rockford, IL). The NKI-L16 antibody, was a generous gift from Drs. Figdor and Joosten (Radboud University, Netherlands). Polyfect transfection reagent was from Qiagen (Valencia, CA). The BMS-806 compound [57], [58] was synthesized by ChemPacific Corp. (Baltimore, MD). Pronase was purchased from Sigma (St. Louis, MO). The C52L peptide [59] was a gift from Dr. Min Lu (Cornell University). Dynasore, a small molecule inhibitor of dynamin, was purchased from Santa Cruz Biothechnology (Santa Cruz, CA). The pCAGGS plasmids encoding for the full length (gp160) HXB2 Env was provided by Dr. J. Binley (Torrey Pines Institute, CA) [78]. The pTYBE-pCAGGS plasmid encoding for the X4-tropic gp160 from a clinical isolate tybe [54] was a gift from Dr. R. Doms (University of Pennsylvania). The HIV-1 molecular clone lacking *env* (pR8ΔEnv) was obtained from Dr. D. Trono (University of Geneva, Switzerland). The pcRev vector expressing HIV-1 Rev [79] and the pMM310 plasmid expressing BlaM-Vpr [80] were obtained from the NIH ARRRP.

### Production and characterization of viruses

pCAGGS-HXB2-gp160 (3 µg), pR8Δenv (2 µg), pMM310 (3 μg) and pcRev (1 µg) and pCDM8 ICAM-1 or an empty vector (3 µg) were mixed with 300 µl DMEM and 120 µl Polyfect. For generating TYBE pseudoviruses [54], the HXB2 gp160 expression vector was replaced with 4 μg TYBE Env-expressing plasmid (pTYBE-pCAGGS). After incubating for 10 min at room temperature, the mixture was diluted to 1 ml with DMEM with 10% FBS and added to 293T cells grown in a 100 mm dish to 50–70% confluency. Twelve hours after transfection, the medium was changed with 6 ml of fresh DMEM/10% FBS without phenol red. The plate was incubated for 36 h at 37°C, 5% CO_2_. Virus-containing extracellular medium was collected, passed through a 0.45 µm filter, and concentrated by ultracentrifugation onto 20% sucrose cushion (100,000×g, 2 h at 4°C), using the SW41 swinging bucket rotor (Beckman). The amount of virus was determined by p24 ELISA, as described in [81]. Infectious titers on TZM-bl cells were determined by a β-Gal assay, as described in [55].

Fluorescent viruses bearing ICAM-1 were produced as above, except that 2 μg of the HIV-1 Gag-iGFP plasmid expressing internal GFP inserted between matrix and capsid proteins [74] was included in the transfection mixture. Viruses were immobilized on a poly-L-lysine coated 8-chamber coverslip (Lab-Tek) and incubated with anti-ICAM-1 mouse antibody 6.5B5 (Santa Cruz, 4 µg/ml) for 1 h at 4°C, washed and further incubated with anti-mouse antibody conjugated with Cy5 (Biolegend, 4 µg/ml) for 1 h at 4°C. Viruses were washed with PBS and imaged using an Olympus fluorescent IX-71 microscope equipped with a 60x oil-immersion objective and EM-CCD camera (Hamamatsu).

### Western blotting

Twenty µl aliquots of concentrated virus preparation were mixed with 20 µl of SDS-PAGE sample buffer (Bio-Rad) supplemented with 5% β-mercaptoethanol and boiled for 10 min at 95°C. Thirty µl of mixture was then loaded onto a 10% polyacrylamide gel (Bio-Rad). Proteins were transferred onto a nitrocellulose membrane, blocked with 10% Blotting-grade Blocker (Bio-Rad) for 1 h at room temperature, and incubated with anti-gp120 antibody (Fitzgerald, 1∶3000 dilution), anti-ICAM-1 antibody (Santa Cruz, 1∶200 dilution), or anti-HIV sera (NIH ARRRP, 1∶3000) in 5% Blocking-grade Blocker overnight at 4°C. Horseradish peroxidase-conjugated anti-mouse antibody (1∶2500 dilution, GE Healthcare) or horseradish peroxidase-conjugated Protein G (1∶2500) were employed for protein detection using a chemiluminescence reagent from GE Healthcare. The resulting signal was visualized on the Chem-Doc Imager (Bio-Rad).

### Virus binding and internalization assay

PM-1 cells (1·10^5^) were pretreated with anti-LFA-1 antibodies, MEM83 or MEM25 in HBSS in V-bottom 96 well plate (Corning Incorporated, Corning, NY) or after adhering to a poly-L-lysine coated 96-well plate (Corning) for 30 min at 37°C or left untreated. HIV-1 preparations (2–10 ng of p24) containing or lacking ICAM-1 were added to cells on ice. Cells were then incubated either at 4°C or at 37°C for 90 min (HXB2 pseudotypes) or 120 min (TYBE pseudotypes). Alternatively, cells and viruses were centrifuged at 2095×g, 4°C for 30 min and washed with cold medium to remove free viruses. After addition of fresh medium with or without anti-LFA-1 antibodies, infection was initiated by shifting to 37°C and stopped after 90 min by putting the plate on ice. Cells were washed with HBSS at the end of incubation and either lysed immediately with 0.5% Triton-X100 in PBS or incubated with 2 mg/ml pronase for 30 min on ice and washed before adding the detergent. Lysates were analyzed by p24 ELISA. A fraction of lysate was used to determine the total protein content of samples using Micro BCA Protein Assay Kit (Thermo Scientific) and plotted as the total amount of protein per well (Fig. S3).

### Virus-cell fusion

Briefly, 1·10^5^ PM-1 cells were adhered onto poly-L-lysine coated 96 well strip-well plates (Corning Costar) for 30 min 37°C. After unbound cells were removed by washing, poly-lysine was blocked by incubation with HBSS/10% FBS for 15 min at room temperature. Immobilized cells were treated with MEM83 (3 µg/ml) for 30 min at 37°C. Viruses bearing or lacking ICAM-1 (0.5–3 ng of p24) were added to the cells and centrifuged and washed as described above. Fusion was initiated by shifting to 37°C for 90 min (HXB2 pseudotypes) or 120 min (TYBE pseudotypes) in the absence or in the presence of 1 µM C52L (negative control), dynasore (80 µM) or 0.1% DMSO. To measure the kinetics of fusion, BMS-806 (10 µM) or C52L (1 µM) were added after varied times of virus-cell incubation or the plates were placed on ice, as described in [55]. At the end of a 90 min-incubation at 37°C, samples were chilled by briefly placed on ice, loaded with the CCF4-AM substrate (GeneBLAzer *in vivo* Detection Kit, Invitrogen), and incubated overnight at 12°C. The resulting BlaM signal was measured using Synergy HT fluorescence plate reader (Bio-Tek Instr. Germany).

#### Cell Viability Assay

PM-1 cells (1·10^5^) were adhered to poly-L-lysine coated strip-well plates, treated with 1 µM C52L, 0.1% DMSO, or 80 µM dynasore in HBSS on ice, and incubated at 37°C for 90 min. At the end of incubation, cells were chilled on ice, overlaid with 50 µl of DMEM/10% FBS and 10 µl of CellTiter 96 Aqueous Reagent (Promega), and further incubated at 37°C for 60 min. The absorbance at 490 nm well was then recorded using Synergy HT fluorescence plate reader.

## Supporting Information

Figure S1 **ICAM-1 facilitates HXB2 pseudovirus binding and fusion with CEM cells.** (A) CEM cells (1·10^5^) were adhered to a poly-lysine-coated 96-well plate and incubated with ICAM^+^ or ICAM^-^ viruses (5 ng of p24) for 1.5 h at 37°C. Free viruses were removed by washing, and the amount of cell-associated particles in each well was determined by p24 ELISA. (B) ICAM^−^ and ICAM^+^ pseudoviruses (1 ng of p24) were incubated with cells at 37°C for 1.5 h, and the extent of fusion was measured by the BlaM assay. Data are means and SEM from a representative experiment done in triplicate.
(TIFF)

Figure S2 **Specific infectivity of TYBE pseudoviruses is not significantly altered by incorporation of ICAM-1.** ICAM^+^ or ICAM^−^ pseudoviruses bearing TYBE Env (0.4 ng of p24) were produced as described in Materials and Methods. Virus titer was determined on TZM-bl cells by the β-Gal assay.
(TIFF)

Figure S3 **The total protein content in PM-1 cell samples does not change significantly following the pronase treatment.** For details, see Materials and Methods and the legend to Figure 3B. A fraction of cell lysate used for the p24 ELISA assay was set aside and the total protein content of these samples was determined by Micro BCA Protein Assay Kit (Thermo Scientific). Data are means and SEM from a representative experiment done in triplicate.
(TIFF)
