# Supplementary figures and images for: Intercellular Adhesion Molecule 1 Promotes HIV-1 Attachment but Not Fusion to Target Cells
Source: PLoS One. 2012 Sep 6;7(9):e44827. doi: 10.1371/journal.pone.0044827 (PMC3435301; doi:10.1371/journal.pone.0044827)

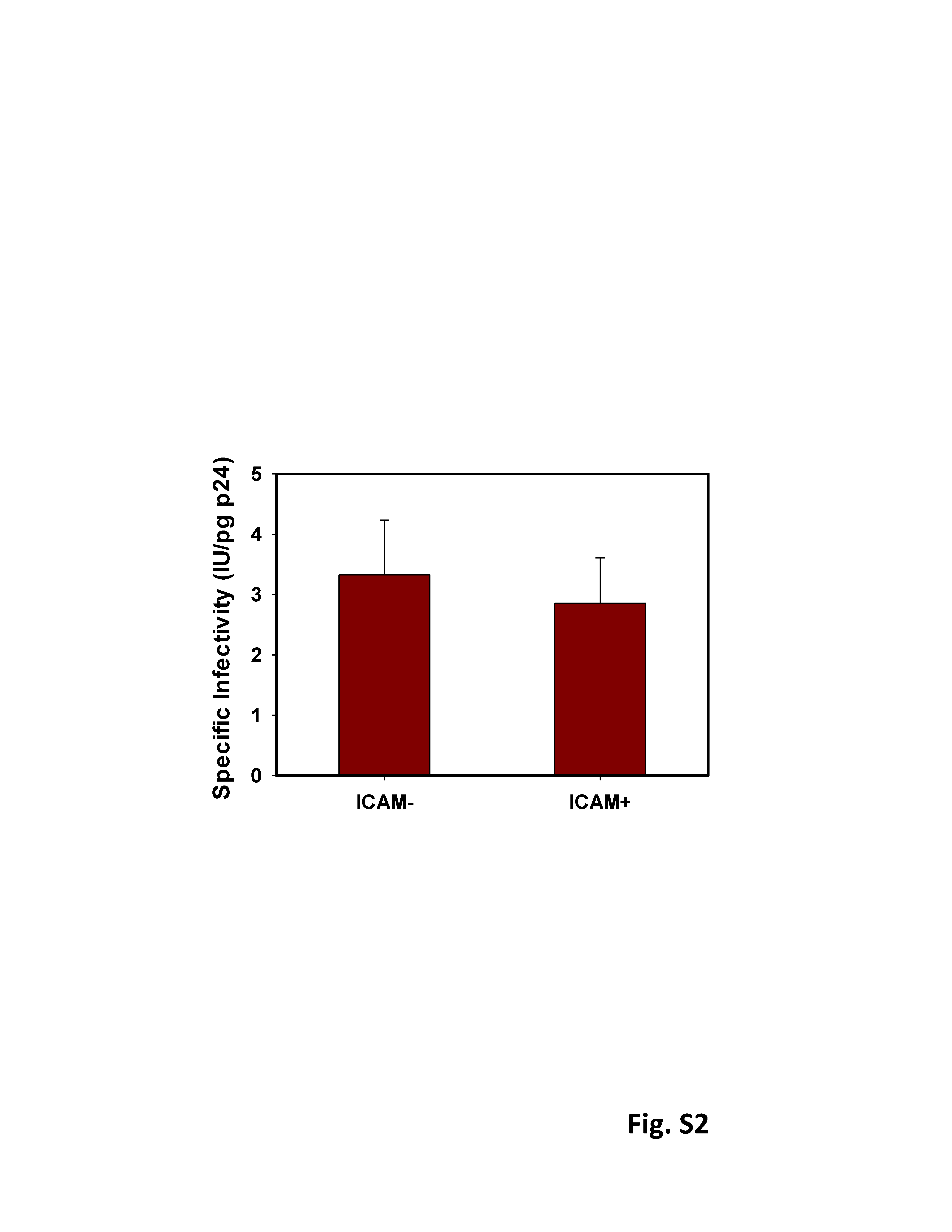

Supplement: Figure S2 — Specific infectivity of TYBE pseudoviruses is not significantly altered by incorporation of ICAM-1. ICAM+ or ICAM− pseudoviruses bearing TYBE Env (0.4 ng of p24) were produced as described in Materials and Methods. Virus titer was determined on TZM-bl cells by the β-Gal assay. (TIFF) [file pone.0044827.s002.tiff]

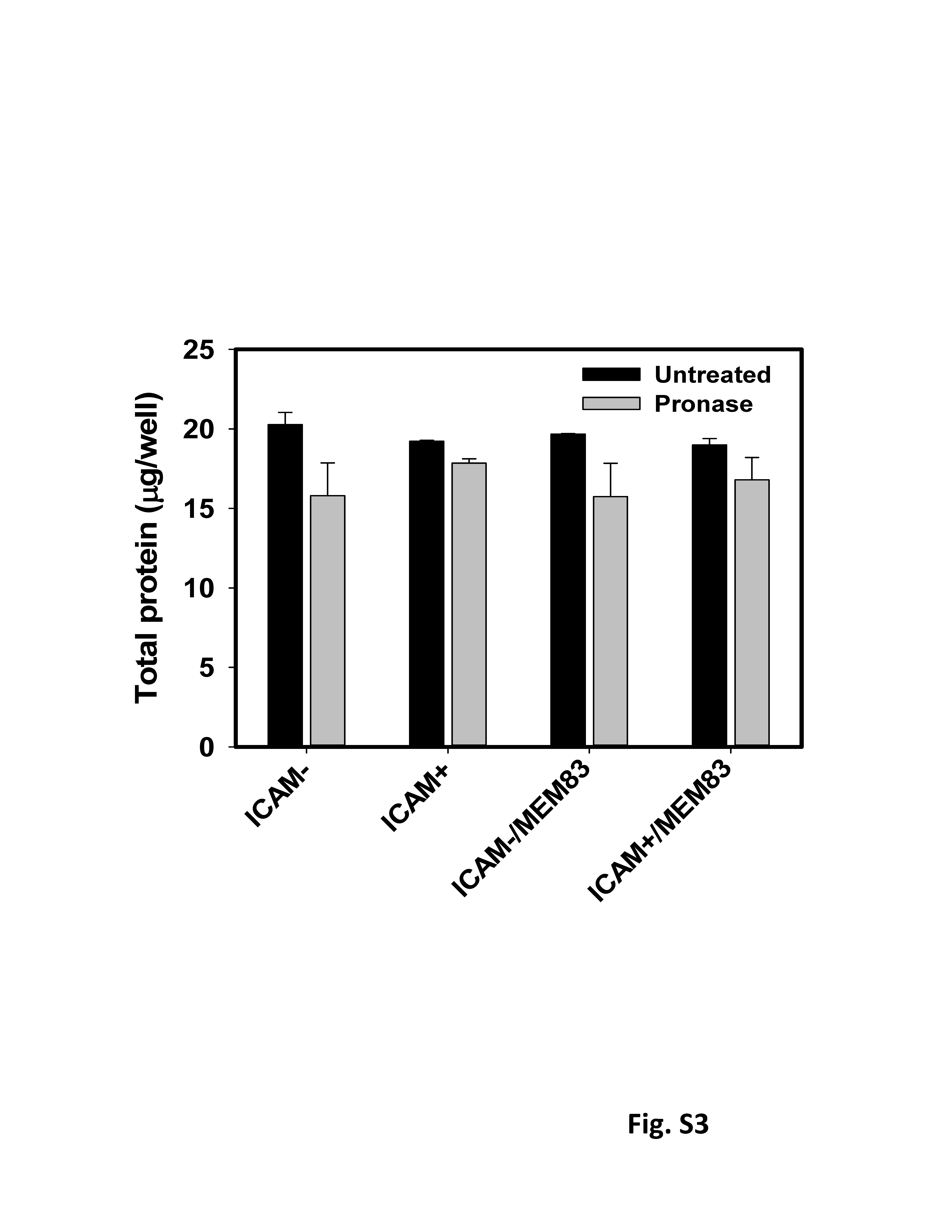

Supplement: Figure S3 — The total protein content in PM-1 cell samples does not change significantly following the pronase treatment. For details, see Materials and Methods and the legend to Figure 3B. A fraction of cell lysate used for the p24 ELISA assay was set aside and the total protein content of these samples was determined by Micro BCA Protein Assay Kit (Thermo Scientific). Data are means and SEM from a representative experiment done in triplicate. (TIFF) [file pone.0044827.s003.tiff]
